# Effect of optimal sodium stearoyl-2-lactylate supplementation on growth performance and blood and carcass characteristics in Hanwoo steers during the early fattening period

**DOI:** 10.5713/ajas.18.0349

**Published:** 2018-07-26

**Authors:** Shin Ja Lee, Sang Suk Lee, Eun Tae Kim, Jin Suk Jeong, Ji Hoon Lee, Joon Jeong, Joong Kook Park, Beom Young Park, Ha Yeon Jeong, Kwang Seok Ki, Chang Hyun Kim, Sung Sill Lee

**Affiliations:** 1 Institute of Agriculture and Life Science and University-Centered Labs, Gyeongsang National University, Jinju 52828, Korea; 2 Ruminant Nutrition and Anaerobe Laboratory, Department of Animal Science and Technology, College of Bio-industry Science, Sunchon National University, Suncheon 57922, Korea; 3 National Institute of Animal Science, RDA, Cheonan 31000, Korea; 4 Division of Applied Life Science (BK21 program) and Institute of Agriculture and Life Science (IALS), Gyeongsang National University, Jinju 52828, Korea; 5 Livestock Research Center, National Agricultural Cooperative Federation, Ansung 17558, Korea; 6 School of Animal Life and Environment Science, Hankyong National University, Ansung 17579, Korea

**Keywords:** Blood Characteristic, Carcass Characteristic, Early Fattening Period, Growth Performance, Sodium stearoyl-2-lactylate

## Abstract

**Objective:**

This study was conducted to evaluate the effect of different levels of total digestible nutrients (TDN) and sodium stearoyl-2-lactylate (SSL) supplementation on growth performance and blood and carcass characteristics in Hanwoo steers during the early fattening period.

**Methods:**

Sixty Hanwoo steers (average body weight, 333±36.4 kg) were randomly allotted to 3 treatments, with twenty steers per treatment, and ten steers per pen with a size of 80 m^2^. Dietary treatments were as follows: CON, basal diet; treatment (TRT) 0.5, 0.5% down-spec of TDN with 0.1% SSL; TRT 1.0, 1.0% down-spec of TDN with 0.1% SSL.

**Results:**

The results demonstrated that average daily gain and feed efficiency increased with TRT 0.5 (0.85 kg and 11.68) vs CON (0.82 kg and 11.27) or TRT 1.0 (0.78 kg and 10.74), indicating that 0.1% SSL supplementation in the feed of early fattening steers may result in a saving of 0.5% TDN. No significant differences were observed amongst all treatments (p> 0.05) for blood metabolite concentration and blood corpuscle values, which were all within the normally accepted range for healthy steers.

**Conclusion:**

Our study suggests that a TDN 0.5% down spec with 0.1% SSL supplemented feed may be effective and profitable for the early fattening period of Hanwoo steers without causing adverse effects.

## INTRODUCTION

Hanwoo steers are usually fattened until almost 30 months and are normally fed high-density concentrate diets from the fattening period onwards. This is to encourage a high degree of marbling, as suggested by Lee et al [1], who reported that Hanwoo steers distinctly improved their marbling between 12 and 27 months of age. Feeding of high-density concentrate diets is used to improve meat quality with high marbling, which is one of the main distinguishing factors determining Hanwoo quality grade [2]. However, feeding of high-density concentrate diets can have a negative effect on digestive metabolism, feed efficiency, and feed intake during the final fattening period [3]. To address these questions, various studies have been carried out on supplementation of emulsifiers in diet [4,5].

Dietary lipid is the nutrient with the highest energy density and needs to be broken down to be readily and efficiently absorbed by the small intestine [6], thus improving dietary lipid digestibility, palatability of the diet and fat yield of cattle [7]. To improve absorption and digestion of lipids, oil in water system surfactants like sodium stearoyl lactylate (SSL) are more effective emulsifiers (hydrophilic lipophilic balance, 10 to 12), than water-in-oil system surfactants like lecithin, in resolving the immiscibility of oil and water and to provide stability to an oil/water system [8].

Previous studies have explored different ways of improving lipid utilization [9,10], more specifically, surfactant supplementation for emulsification purposes in ruminants [11]. For example, the beneficial effects of surfactants have been confirmed in ruminal microbial growth rates [10,12], nutrient digestibility [13,14], feed intake [12,15], growth performance [5,16], and ruminal enzyme activity and accessibility [12,17]. However, responses to surfactants have not always shown positive effects on ruminal fermentation and growth performance [18]. As such, the present study was conducted to investigate the efficiency of 0.1% SSL supplementation with different levels of total digestible nutrients (TDN, 0.5% or 1.0% reduction) on growth performance and blood and carcass characteristics in Hanwoo steers during the early fattening period.

## MATERIALS AND METHODS

All experimental protocols used in this study were approved by the Animal Care Committee of Gyeongsang National University (Jinju, Gyeongsangnam-do, Republic of Korea).

### Chemicals

SSL is composed of lactic acid mono-esters with sodium salt and its IUPAC (International Union of Pure and Applied Chemistry) chemical name is isoctadecanoic acid, 2-(1-carbocyethoxy)-1-methyl-2-oxoethyl ester, sodium salt. SSL has the molecular formula C_24_H_43_O_6_Na (MW, 450.58 g/mol), and is a versatile organic compound with several qualities as a food additive ; it improves the volume and mix tolerance of processed foods and is approved by the FDA [19]. Having both a hydrophilic head solving water and lipophilic tail solving oil, SSL can decrease interfacial tension, permitting a more stable state. Additionally, SSL is biodegradable [20], non-toxic [21], and found in typically biorenewable additives ranging from mixes, baked goods, processed goods, dairy products and pet foods [22].

### Animals, diets and managements

The experiment was conducted for 200 days at the National Agricultural Cooperative Federation Farm located in Anseong, South Korea. A total of sixty Hanwoo steers (13 months old, average body weight [BW] 333±36.4 kg) were used in a completely randomized design and allotted to 3 treatment groups, with twenty steers per treatment and ten steers per pen with a size of 80 m^2^ (8×10 m) as shown in Table 1. Dietary treatments were as follows: CON, basal diet; treatment (TRT) 0.5, 0.5% down spec of TDN (72.02%) with 0.1% SSL; TRT 1.0, 1.0% down spec of TDN (71.53%) with 0.1% SSL. The experiment was carried out on animals from 13 to 20 months of age. All groups received concentrate and rice straw bale throughout the feeding trial (1 to 60 days, 4.86 kg of commercial concentrate and 1.80 kg of rice straw bale; 61 to 120 days, 5.86 kg of commercial concentrate and 1.35 kg of rice straw bale; 121 to 200 days, 6.75 kg of commercial concentrate and 0.90 kg of rice straw bale). The ingredient composition and chemical analysis of the concentrate diets are shown in Table 2. Steers were fed the experimental diets three times daily at 07:30, 13:00, and 18:00 in their stalls and diets were supplemented with SSL consisting of the top dressing of each treatment ration.

After a 7 day adaptation period, animals were exposed to different treatments during the whole experimental period. Animals were provided with access to water and mineral blocks *ad libitum* and placed in an environmentally regulated facility throughout the 200 days of the experimental period. The typical feeding management standard for beef cattle in Korea was followed.

### Diet chemical analysis

Experimental diet samples were dried in a forced-air oven at 130°C for 2 h, then finely ground to a size that could pass through a 2 mm sieve in a Wiley mill (Model 4, Thomas Scientific, Swedesboro, NJ, USA). The ground samples were analyzed for dry matter (Method 930.15), crude protein (Method 984.13), Ca (Method 984.01), and P (Method 965.17) according to AOAC procedures. Ether extraction was measured by the diethyl ether extraction method using a Buchi B-811 Universal Extraction System (Buchi, Flawil, Switzerland); crude fiber was analyzed by the filter bag technique using the ANKOM 220 Fiber Analyzer (Mill tech, Seongnam, Republic of Korea).

### Physical and carcass traits

The BW was recorded individually at the beginning of the experiment, after a 2-month interval, and at the end of the experiment. Weight gain was calculated the difference between initial and final BW, and feed efficiency by dividing BW by total feed intake.

Carcass traits were measured by ultrasound and included: backfat thickness (UBF), eye muscle area (UEMA), and marbling score (UMS), which are three important economic values according to characteristics graded by the Korean Institute for Animal Products Grading Service. For ultrasound image measurements, a Real-time B mode HS-2000 (FHK Co., Tokyo, Japan) with an attached 2.0 MHz Linear probe was used. Ultrasound scanning was on done the left side between the 13th rib and the first lumbar vertebrae. UEMA and UBF were calculated by a devoted ultrasound image analysis program and UMS was marked as a score in a range of 1 to 27 based on the subjective judgment of experts. Meat quantity was classified on a scale of A, B, and C, where A is the highest and C the lowest yield; meat quality grade was scored on a scale of 1++, 1+, 1, 2, and 3 by trained personnel from the Animal Products Grading Service (Seoul, Republic of Korea).

### Blood sampling

Blood samples were obtained by direct venipuncture of the jugular vein at the end of the experimental period, prior to the morning feeding. Whole blood samples (3 mL) were collected into 10 mL BD vacuum tubes with sodium heparin (Becton and Dickinson, Franklin Lakes, NJ, USA). After allowing to clot at 4°C for 24 h, samples for serum analysis were centrifuged at 3,000 rpm for 10 min at 4°C, and then separated and stored at −70°C. Blood samples were analyzed using a Hitachi 7020 automatic blood analyzer (Hitachi, Tokyo, Japan) for total protein, phosphorus, albumin, total bilirubin, cholesterol, aspartate aminotransferase (AST), alanine aminotransferase (ALT), Ca, gamma glutamyl transferase, blood urea nitrogen (BUN), creatinine, total triglycerides, non-esterified fatty acid, and glucose.

Whole blood samples were used to measure hematological parameters including red blood cell (RBC), hematocrit (HCT), hemoglobin (HGB), mean corpuscular volume, mean corpuscular hemoglobin, mean corpuscular hemoglobin concentration, white blood cell (WBC), neutrophil (NEU), lymphocyte (LYM), monocyte (MONO), eosinophil, basophil, and platelet counts.

### Statistical analysis

All data for steers within each treatment were averaged and analyzed using the PRO GLM procedure of the SAS statistical program, package ver. 9.1 (2005; SAS Inst. Inc., Cary, NC, USA) with the statistical model of Y_ij_ = μ+TRT_i_+e_ij_, where Y_ij_ is an observation on the dependent variable ij, μ is the overall population mean, TRT_i_ is the fixed effect of treatments, and e_ij_ was the random error associated with the observation ij. Duncan’s multiple range test was used to identify any significant differences among the mean values of the treatments. Variability in the data was expressed as the standard error of the mean, and p<0.05 was considered statistically significant, whereas p<0.10 was considered a tendency.

## RESULTS AND DISCUSSION

### Growth performance

This experiment was conducted to evaluate the effect of different levels of TDN (CON, 72.54%; TRT 0.5, 72.2%; TRT 1.0, 71.5%) and SSL supplementation (TRT 0.5 and TRT 1.0; additional SSL 0.1%) on growth performance in Hanwoo steers. Throughout the experiment, a major source of the diet (i.e. concentrates and forages) supplied to the steers was restricted and others were similar among different treatments. Therefore, feed intakes divided into three periods are presented as mean values: each total feed intake was 6.66 kg DM/d (4.86 kg of concentrate and 1.8 kg of forage) for days 0 to 60; 7.20 kg DM/d (5.85 kg of concentrate and 1.35 kg of forage) for days 61 to 120; and 7.65 kg DM/d (6.75 kg of concentrate and 0.9 kg of forage) for days 121 to 200, according to a commercially used early fattening program for Hanwoo steers.

As shown in Table 3, the final body weight for TRT 0.5 (392.4, 442.1, and 502.1 kg) increased as compared with CON (385.5, 439.9, and 496.8 kg) and TRT 1.0 (376.3, 429.1, and 487.7 kg) in all three periods; however, the difference was not significant (p>0.05) indicating that SSL supplementation had no negative effect. Our results are in agreement with Jeong et al [5] who reported that TDN down spec with SSL 0.03% supplementation showed no significant differences in average daily gain and final body weight in the final fattening period of Hanwoo steers as compared with CON. Why the different TDN levels with 0.1% SSL supplementation had no significant negative effect on final body weight may be that supplemented non-ionic surfactants can enhance ruminal fermentation and improve feed utilization efficiency, which can be attributed to stimulating effects of non-ionic surfactants on growth performance, as demonstrated by Lee et al [17] and Wang et al [15]. Furthermore, Nylander and Wang [8] demonstrated that SSL is an extremely efficient at facilitating the formation of fat-in-water emulsions for lipid digestion in the small intestine and can therefore lead to improved growth performance. However, TRT 1.0 (TDN 1.0% down spec with SSL 0.1%) was shown to reduce body weight as compared with CON and TRT 0.5 (TDN 0.5% down spec with SSL 0.1%). During the early fattening period, steers need relatively high levels of TDN in their diets to support normal and sustained growth [23,24] and, even a 1% lower TDN may significantly affect growth performance despite SSL supplementation.

Feed efficiency, which results in either greater body weight or less feed intake [25], improved with TRT 0.5 (15.12 and 10.03) as compared with CON (13.30 and 9.42) and TRT 1.0 (11.35 and 9.70) at 1 to 60 and 121 to 200 days, but was not statistically significant (p>0.05) as shown in Table 3. Nonetheless, SSL supplementation in Hanwoo steers improved feed efficiency, which is in agreement with previous studies by Jeong et al [5], who showed that TDN down spec with SSL 0.03% supplementation had no significant negative effect on feed efficiency when compared to controls.

In general, the purpose of adding appropriate fats or oils to the feed of high-producing steers, in the early fattening period is to supply available energy for the rumen microbes resulting in improvements in feed efficiency and growth performance [26]. Consequently, average daily gain and feed efficiency in the whole early fattening period of TRT 0.5 (0.85 and 11.68 kg) was higher than CON (0.82 and 11.27 kg) or TRT 1.0 (0.78 and 10.74 kg), suggesting that a 0.1% SSL supplementation in the feed of early fattening steers can potentially result in a net a saving of 0.5% TDN without any negative effect.

### Blood characteristics

Blood metabolite concentrations and blood corpuscle of early fatting steers supplemented with 0.1% SSL and different levels of TDN down spec (0.5% and 1.0%) are shown in Table 4 and 5, Blood metabolite concentrations is considered a useful indicator for monitoring nutrient status, and body condition, for prevention of disease [27,28], and to evaluate the internal metabolic changes and the function of different organs in cattle, including the kidneys and liver. No significant differences were observed amongst all treatments (p>0.05) and the values of all blood metabolite concentrations were all within the normally accepted ranges for healthy steers, as suggested by Alex [29]. In particular, the levels of albumin, creatine, BUN, AST, and ALT, all associated with liver and tissue damage, did not show any significant differences, indicating that different levels of TDN down spec (0.5% and 1.0%) with 0.1% SSL supplementation did not produce any negative physiological effects when compared to CON.

The most common blood diagnostic test is the determination of blood count which includes RBC, HCT, HGB, WBC, NEU, LYM, and MONO. In the current study, no statistical differences were observed amongst all treatments (p>0.05), except with NEU, LYM, and MONO as shown in Table 5. An interesting tendency was observed, with TRT 0.5 stimulating NEU (3.66 K/μL) while the LYM (5.37 K/μL) and MONO (0.58 K/μL) were depressed as compared with CON (3.59, 5.80, and 0.64 K/μL) and TRT 1.0 (3.04, 6.28 and 0.73 K/μL), respectively (p<0.10). However, the values of all these measures were all within the normally accepted range, as suggested by Alex [29], indicating that no significant changes were induced by the different levels of TDN down spec (0.5% and 1.0%) and 0.1% SSL supplementation.

### Carcass characteristics

Carcass yield and quality traits of early fattening steers supplemented with 0.1% SSL and different levels of TDN downspec (0.5% and 1.0%) are shown in Table 6. Carcass yield decreased with TRT 0.5 (UBF, 4.62 mm and UEMA, 62.44 cm^2^) as compared with CON (UBF, 5.33 mm and UEMA, 63.45 cm^2^) and TRT 1.0 (UBF, 5.45 mm and UEMA, 64.04 cm^2^); however, no significant differences were observed amongst all the treatments (p>0.05). Value for meat quality traits also decreased with TRT 0.5 (UMS, 3.00, meat quantity grade, 2.70 and meal quality grade 1.30) as compared with CON (UMS, 3.60, meat quantity grade, 2.60 and meal quality grade 1.40) and TRT 1.0 (UMS, 3.50, meat quantity grade, 2.70 and meal quality grade 1.40); again, no significant differences were observed amongst all the treatments (p>0.05).

Realini et al [30] have suggested that backfat thickness increases noticeably in the final fattening period, from the growing period to 14 days before slaughter. Additionally, Kim [31] suggested that backfat thickness of Hanwoo steers generally develops rapidly, after the steers had attained 500 kg BW. As research was conducted during the early fattening period when the average BW of steers ranged from 332.63 to 495.53 kg, further studies are needed to better understand how TDN downspec and SSL supplementation can subsequently influence carcass yield and quality.

## CONCLUSION

In summary, in this study we demonstrated that average daily gain and feed efficiency improved with TRT 0.5 as compared to CON and TRT 1.0 and without causing any adverse effects, implying that 0.1% SSL supplementation in the feed of early fattening steers may potentially result in a saving of 0.5% TDN. With respect to blood and carcass characteristics, no significant differences were observed amongst all treatments (p>0.05). The values of all blood parameters were within the normally accepted range for healthy steers. Therefore, SSL supplementation with TND downspeccing appears to be a viable feed cost-saving measure for fattening of Hanwoo steers, without any adverse effects. However, further studies are needed to better understand the effect of TND down-specing and SSL supplementation on growth performance and blood and carcass characteristics, in both the early and final fattening periods of Hanwoo steers.

## Figures and Tables

**Table 1:** Animal characteristics and dietary treatments

Item	Treatment1)		

	CON	TRT 0.5	TRT 1.0
No. of heads	20	20	20
Body weight (kg)	333.2±45.0	333.0±28.1	331.7±36.1
Age (month)	13.7±0.7	13.8±0.8	13.5±1.0
Castration (month)	8.5	8.5	8.5

CON, control; TRT, treatment; TDN, total digestible nutrients; SSL, sodium stearoyl-2-lactylate.

1) CON, basal diet; TRT 0.5, 0.5% downspec of TDN with 0.1% addition of SSL; TRT 1.0, 1.0% downspec of TDN with 0.1% addition of SSL.

**Table 2:** Experimental diet formulas

Item	Treatment1)		

	CON	TRT 0.5	TRT 1.0
	------------------------ Formula, % -----------------------		
Maize	28.1	25.1	25.4
Wheat grain	11.0	11.0	10.0
Cane molasses	4.5	4.5	5.0
Tapioca residue	7.0	8.6	12.0
Wheat bran	2.0	2.0	2.0
Corn gluten feed	19.1	20.0	14.0
Cottonseed	3.0	3.0	3.3
Soybean oil	0.1	0.1	0.1
Coconut meal	11.0	11.0	11.0
Palm kernel meal	11.0	11.0	11.0
Salt dehydrated	0.5	0.5	0.5
Limestone, 1 mm	1.6	2.5	2.3
Calcium sulfate	0.2	0.2	0.2
Vitamin premix	0.1	0.1	0.1
Mineral premix	0.1	0.1	0.1
SSL	-	0.1	0.1
	----------------- Chemical composition, % -------------		
TDN	72.54	72.02	71.53
Dry matter	88.82	88.51	88.24
Crude protein	12.20	12.18	12.20
Ether extract	3.67	3.62	3.48
Crude fiber	7.88	8.05	8.25
Ca	0.80	1.16	1.13
P	0.39	0.39	0.37
Crude ash	6.27	7.30	7.23

CON, control; TRT, treatment; SSL, sodium stearoyl-2-lactylate; TDN, total digestible nutrients.

1) CON, basal diet; TRT 0.5, 0.5% downspec of TDN with 0.1% addition of SSL; TRT 1.0, 1.0% downspec of TDN with 0.1% addition of SSL.

**Table 3:** Effects of sodium stearoyl-2-lactylate (SSL) supplementation on growth performance during the early fattening stage in Hanwoo steers

Item	Treatment1)			SEM	p-value

	CON	TRT 0.5	TRT 1.0		
	---------- 1 to 60 days --------				
Final body weight (kg)	385.5	392.4	376.3	4.84	0.4015
Average daily gain (kg)	0.89	1.01	0.76	0.05	0.1405
DM intake, concentrate	4.86	4.86	4.86	-	-
rice straw	1.80	1.80	1.80	-	-
total	6.66	6.66	6.66	-	-
Feed efficiency	13.30	15.12	11.35	0.76	0.1410
	--------- 61 to 120 days --------				
Final body weight (kg)	439.9	442.1	429.1	5.07	0.5362
Average daily gain (kg)	0.89	0.81	0.87	0.02	0.3065
DM intake, concentrate	5.85	5.85	5.85	-	-
rice straw	1.35	1.35	1.35	-	-
total	7.20	7.20	7.20	-	-
Feed efficiency	12.40	11.32	12.02	0.29	0.3209
	-------- 121 to 200 days -------				
Final body weight (kg)	496.8	502.1	487.7	5.71	0.5905
Average daily gain (kg)	0.72	0.77	0.74	0.02	0.6039
DM intake, concentrate	6.75	6.75	6.75	-	-
rice straw	0.90	0.90	0.90	-	-
total	7.65	7.65	7.65	-	-
Feed efficiency	9.42	10.03	9.70	0.24	0.5960

CON, control; TRT, treatment; SEM, standard error of the mean; DM, dry matter; TDN, total digestible nutrients.

1) CON, basal diet; TRT 0.5, 0.5% downspec of TDN with 0.1% addition of SSL; TRT 1.0, 1.0% downspec of TDN with 0.1% addition of SSL.

**Table 4:** Effects of sodium stearoyl-2-lactylate (SSL) supplementation on blood metabolite values during the early fattening stage in Hanwoo steers

Item	Treatment1)			SEM	p-value

	CON	TRT 0.5	TRT 1.0		
Total protein (g/dL)	6.29	6.23	6.19	0.07	0.5216
Phosphorus (mg/dL)	7.93	7.72	7.45	0.06	0.2514
Albumin (g/dL)	3.53	3.46	3.41	0.03	0.1432
Total bilirubin (mg/dL)	0.28	0.27	0.27	0.004	0.5389
Cholesterol (mg/dL)	146.21	155.58	166.50	1.96	0.3204
AST (IU/L)	67.08	64.67	64.73	0.44	0.2956
ALT (IU/L)	22.26	23.23	23.35	0.22	0.1254
Ca (mg/dL)	4.43	4.62	4.71	0.08	0.1977
GGT (mg/dL)	21.35	21.75	23.56	0.32	0.1538
BUN (mg/dL)	10.07	10.96	9.93	0.14	0.2548
Creatine (mg/dL)	1.19	1.15	1.16	0.02	0.2648
Total glyceride (mg/dL)	23.47	23.25	22.75	0.21	0.3512
NEFA (μEq/L)	199.68	166.28	141.23	4.63	0.4155
Glucose (mg/dL)	117.29	108.33	109.90	1.23	0.1258

CON, control; TRT, treatment; SEM, standard error of the mean; AST, aspartate aminotransferase; ALT, alanine aminotransferase; GGT, gamma glutamyl transferase; BUN, blood urea nitrogen; NEFA, non-esterified fatty acid; TDN, total digestible nutrients.

1) CON, basal diet; TRT 0.5, 0.5% downspec of TDN with 0.1% addition of SSL; TRT 1.0, 1.0% downspec of TDN with 0.1% addition of SSL.

**Table 5:** Effect of sodium stearoyl-2-lactylate (SSL) supplementation on blood corpuscle values during the early fattening stage in Hanwoo steers

Item	Treatment1)			SEM	p-value

	CON	TRT 0.5	TRT 1.0		
RBC (M/μL)	8.26	8.70	7.86	0.16	0.2151
HCT (%)	34.40	36.76	35.10	0.29	0.1263
HGB (g/dL)	12.13	12.83	12.15	0.07	0.1684
MCV (fL)	41.69	42.33	44.75	0.33	0.2084
MCH (pg)	14.71	14.78	15.50	0.18	0.1634
MCHC (g/dL)	35.31	34.94	34.69	0.14	0.2441
WBC (K/μL)	10.61	10.51	11.17	0.14	0.1642
NEU (K/μL)	3.59	3.66	3.04	0.03	0.0954
LYM (K/μL)	5.80	5.37	6.28	0.24	0.0864
MONO (K/μL)	0.64	0.58	0.73	0.03	0.0674
EOS (K/μL)	0.86	0.86	1.08	0.03	0.1251
BASO (K/μL)	0.02	0.01	0.03	0.001	0.1254
PLT (K/μL)	156.61	208.23	204.50	3.92	0.2634

CON, control; TRT, treatment; SEM, standard error of the mean; RBC, red blood cell; HCT, hematocrit; HGB, hemoglobin; MCV, mean corpuscular volume; MCH, mean corpuscular hemoglobin; MCHC, mean corpuscular hemoglobin concentration; WBC, white blood cell; NEU, neutrophil; LYM, lymphocyte count; MONO, monocyte; EOS, eosinophil; BASO, basophil; PLT, platelet count; TDN, total digestible nutrients.

1) CON, basal diet; TRT 0.5, 0.5% downspec of TDN with 0.1% addition of SSL; TRT 1.0, 1.0% downspec of TDN with 0.1% addition of SSL.

**Table 6:** Effect of sodium stearoyl-2-lactylate (SSL) supplementation on yield and quality traits during the early fattening stage in Hanwoo steers

Item	Treatment1)			SEM	p-value

	CON	TRT 0.5	TRT 1.0		
	------------- Initial period ---------				
UBF (mm)	2.74	2.69	2.66	0.10	0.984
UEMA (cm^2^)	48.14	49.04	48.84	0.63	0.913
UMS (No. 1 – 9)	1.00	1.00	1.00	-	-
Meat quantity grade2)	3.00	3.00	3.00	-	-
Meat quality grade3)	1.00	1.00	1.00	-	-
	------------- Final period ----------				
UBF (mm)	5.33	4.62	5.45	0.21	0.461
UEMA (cm^2^)	63.45	62.44	64.04	0.69	0.692
UMS (No. 1 – 9)	3.60	3.00	3.50	0.26	0.538
Meat quantity grade2)	2.60	2.70	2.70	0.06	0.825
Meat quality grade3)	1.40	1.30	1.40	0.06	0.783

CON, control; TRT, treatment; SEM, standard error of the mean; UBF, ultrasound backfat thickness; UEMA, ultrasound eye muscle area; UMS, ultrasound marbling score; TDN, total digestible nutrients.

1) CON, basal diet; TRT 0.5, 0.5% downspec of TDN with 0.1% addition of SSL; TRT 1.0, 1.0% downspec of TDN with 0.1% addition of SSL.

2) Meat quantity grade (A grade = 3; B grade = 2; C grade = 1).

3) Meat quality grade (2 to 3 grade = 1; 1 to 2 grade = 2; 1+ to 1++ grade = 3).

## References

[1] Lee SH, Park EW, Cho YM (2007). Identification of differentially expressed genes related to intramuscular fat development in the early and late fattening stages of Hanwoo steers. J Biochem Mol Biol.

[2] Lee DH (2004). Methods of genetic parameter estimations of carcass weight, longissimus muscle area and marbling score in Korean cattle. J Anim Sci Technol (Kor).

[3] Lee SM, Kim JY, Kim EJ (2012). Effects of stocking density or group size on intake, growth, and meat quality of Hanwoo steers (*Bos taurus coreanae*). Asian-Australas J Anim Sci.

[4] Goto M, Bae H, Lee SS (2003). Effects of surfactant Tween 80 on forage degradability and microbial growth on the *in vitro* rumen mixed and pure cultures. Asian-Australas J Anim Sci.

[5] Jeong J, Hwang JM, Seong NI (2009). Effects of supplemented PROSOL^®^ as an emulsifier on growth performance and carcass characteristics in Hanwoo steers of final fattening period. Kor J Anim Sci Technol.

[6] Kim YY, Ha JK, Han IK (2009). Animal nutrition.

[7] Cho HT, Salvia-Trujillo L, Kim J (2014). Droplet size and composition of nutraceutical nanoemulsions influences bioavailability of long chain fatty acids and Coenzyme Q10. Food Chem.

[8] Nylander G, Wang Z, Hasenhettl GL, Hartel RW (2010). Guidelines for processing emulsion-based foods.

[9] Akin DE (1980). Evaluation by electron microscopy and anaerobic culture of types of rumen bacteria associated with digestion of forage cell walls. Appl Environ Microbiol.

[10] Lee SS, Ha JK (2003). Influences of surfactant Tween 80 on the gas production, cellulose digestion and enzyme activities by mixed rumen microorganism. Asian-Australas J Anim Sci.

[11] Ahn GC, Kim JH, Park EK (2009). Effects of non-ionic surfactant supplementation on ruminal fermentation, nutrient digestibility and performance of beef steers fed high-roughage diets. Asian-Australas J Anim Sci.

[12] Kim WJ, Gamo Y, Sani YM (2006). Effect of Tween 80 on hydrolytic activity and substrate accessibility of carbohydrolase I (CBH I) from Trichoderma viride. Asian-Australas J Anim Sci.

[13] Dersjant-Li YM, Peisker M (2005). Soybean lecithin in animal nutrition: an unmatched additive. Kraftfutter.

[14] Hess BW, Moss GE, Rule DC (2008). A decade of developments in the area of fat supplementation research with beef cattle and sheep. J Anim Sci.

[15] Wang Y, Greer D, McAllister TA (2003). Effects of roller setting and saponin based surfactant on barley processing, ruminal degradation of barley, and growth performance by feedlot steers. J Anim Sci.

[16] Jin CF, Kim JH, Han IK, Jung HJ, Kwon CH (1998). Effects of various fat sources and lecithin on the growth performances and nutrient utilization in pigs weaned at 21 days of age. Asian-Australas J Anim Sci.

[17] Lee SS, Ahn BH, Kim HS (2003). Effects of non-ionic surfactants on enzyme distributions of rumen contents, anaerobic growth of rumen microbes, rumen fermentation characteristics and performance of lactating cows. Asian-Australas J Anim Sci.

[18] Hristov AN, McAllister TA, Olson ME (2000). Effect of Tween 80 and salinomycin on ruminal fermentation and nutrient digestion in steers fed a diet containing 70% barley. Can J Anim Sci.

[19] FDA (2003). Toxicological principles for the safety assessment of food ingredients, center for food safety and applied nutrition.

[20] Schaefer EC, Matthews ME (2007). Fatty acids, C16-18 and C18-unsaturated, reaction products with lactic acid and monosodium lactate (CAS# 847904-46-5): ready biodegradability by the carbon dioxide evolution test method, project No.645E-101 for caravan ingredients.

[21] Lamb J, Hentz K, Schmitt D (2010). A one-year oral toxicity study of sodium stearoyl lactylate (SSL) in rats. Food Chem Toxicol.

[22] Ash M, Ash I (2004). Handbook of green chemicals.

[23] Comerford JW, Harpster HW, Baumer VH (2001). The effects of grazing, liquid supplements, and implants on feedlot performance and carcass traits of Holstein steers. J Anim Sci.

[24] Li SG, Yang YX, Rhee YJ (2010). Growth, behavior, and carcass traits of fattening Hanwoo (Korean Native Cattle) steers managed in different group sizes. Asian-Australas J Anim Sci.

[25] Nielsen MK, MacNeil MD, Dekkers JCM (2013). Life-cycle, totalindustry genetic improvement of feed efficiency in beef cattle: Blueprint for the beef improvement federation. Prof Anim Sci.

[26] Choi HS (2013). Effect of supplemented sodium stearoyl lactylate (SSL) on growth performance in Hanwoo steers of final fattening period [PhD thesis].

[27] Payne JM, Dew SM, Manston R, Faulk M (1970). The use of a metabolic profile test in dairy herds. Vet Rec.

[28] Cho HU, Ko WS, Son HW (2008). Hematological and biochemical analysis of Korean indigenous cattle according to the ages. Kor J Vet Serv.

[29] Alex S (2011). Using biological tests to monitor dairy cow fertility, reproductive hormones and other metabolites. Repro. Connections [Internet].

[30] Realini CE, Williams RE, Pringle TD, Bertrand JK (2001). Gluteus medius and rump fat depths as additional live animal ultrasound measurements for predicting retail product and trimmable fat in beef carcasses. J Anim Sci.

[31] Kim W (2009). Early prediction of backfat depth and marbling score of Hanwoo by ultrasound [PhD thesis].

